# Medical Association of Georgia

**Published:** 1873-05

**Authors:** 


### MEDICAL ASSOCIATION OF GEORGIA.

It is a source of congratulation to all who feel an interest in the welfare of the medical profession in this State, that the last meeting, held here last month, was so entirely harmonious, and exhibited such a general and marked interest in the substantial objects of the body. All divisions and wrangling have been effectually *crushed* out, and by the provisions of the new and simplified constitution, all probable sources of difficulty in the future have been removed. While the wise suggestion of Dr. Gross, for the American Medical Association, in regard to a judicial council whose decisions should be final, was fully adopted by that body, we were not able to secure a united action upon this point in the State Association, and it was therefore thought best by the friends of the new measure not to press it for the present; but we are confident that the example of the higher body will have its influence in securing a similar action in the future. A Board of Censors was however appointed, which will to a great extent meet the objects of those who desired to exclude from the body all possibility of discussions not strictly of a scientific character.

While local societies are desirable and encouraged, their delegates to the State Association are no longer entitled to the privileges of members, but must become so, if at all, by being elected in the usual way. It is not in any sense a representative body, but an association of medical men—an annual reunion of the profession for the general advancement of science and practical medicine.

The harmony and good feeling which prevailed at the recent meeting of the Georgia Medical Association, and the large number of papers presented for consideration, prove that physicians are capable of conducting their meetings in a manner to promote the advancement of medical science. It is hoped and believed that the portals are now so guarded that the elements of discord in the form of selfishness, personal pique, etc., can not hereafter find their way into our deliberations.

